# Preparation and Characterization of Simvastatin-Loaded PCL/PEG Nanofiber Membranes for Drug Sustained Release

**DOI:** 10.3390/molecules27217158

**Published:** 2022-10-22

**Authors:** Lu Pan, Jiahao Yang, Lan Xu

**Affiliations:** College of Textile and Engineering, Soochow University, Suzhou 215123, China

**Keywords:** electrospinning, simvastatin, composite nanofiber membranes, drug release

## Abstract

Simvastatin (SIM) particles are liposoluble drugs with large particle sizes, resulting in poor compatibility with electrospun polycaprolactone (PCL)/polyethylene glycol (PEG) nanofibers, so that part of them will be exposed to the electrospun nanofiber surface, which is easy to cause the burst release of drugs. Therefore, in this paper, stearic acid (SA) with good biocompatibility was innovatively added to increase the dispersion uniformity of SIM in the spinning solution, thus improving the performances of SIM-loaded PCL/PEG nanofiber membranes (NFMs). Accordingly, the effects of SA addition on the morphologies, mechanical properties, wettability, and drug release properties of the SIM-loaded NFMs were studied. The results showed that after SIM was dissolved in SA solution, the particle size of SIM was significantly reduced and could be evenly dispersed in the polymer spinning solution, thus obtaining the SIM-loaded composite NFMs with the best morphology and performance.

## 1. Introduction

The controlled/sustained release of drugs can alleviate or avoid the side effects caused by the initial burst release of drugs, stabilize the blood drug concentration, reduce the toxic and side effects of drugs, and improve the stability and safety of drug treatments. Nanotechnology is one of the important means to achieve this goal. Common drug carriers based on nanotechnology include liposomes, micelles, dendrimers, nanotubes, nanoparticles, nanofibers, and nanogels [1]. Among them, nanofibers prepared by electrospinning technology can have significant advantages in controlling the drug release rate by changing nanofiber composition (such as using hydrophobic [2] or hydrophilic materials [3] as drug carriers), nanofiber microstructure (such as smooth [4], porous [5], beaded [6] or core-shell [7] fiber structure) or nanofiber membrane macrostructure (such as sandwich grid structure [8]). In addition, nanofibers as drug carriers must be safe and non-toxic and have good biocompatibility, mechanical properties, and biodegradability [9]. Therefore, nanofibers based on biodegradable natural and synthetic polymers [10] are developing rapidly as drug delivery systems. So far, a large number of biodegradable polymers, such as polyethylene glycol (PEG) [11], chitosan (CS) [12], polylactic acid (PLA) [13], and polycaprolactone (PCL) [14], have been used in electrospinning technology to prepare nanofibers [15], which makes electrospinning technology have a broad development prospect in the field of drug delivery.

Electrospinning is a simple and effective technology for preparing nanofibers, which can control the morphology and structure of nanofibers by adjusting spinning solution components, spinning process parameters, ambient conditions, and post-processing parameters. An electrospinning device is mainly composed of a spinning solution supply device, a spinneret, a high-voltage power supply, and a collector [16]. On applying a high voltage, a Taylor cone is formed at the spinning nozzle. When the electric field force exceeds the surface tension of the Taylor cone, a charged jet is ejected from the tip of the Taylor cone, which is stretched and solidified, finally forming nanofibers to deposit on the collector. The structure and chemical composition of electrospun nanofibers are similar to the natural extracellular matrix, and the high porosity of interconnected fibers can promote the attachment, migration, and proliferation of cells [17], making them have great potential in the biomedical field [18].

In the drug delivery system based on electrospinning, the compatibility of drugs and fiber materials will affect the coating and release of drugs. Accordingly, selecting fiber materials compatible with drugs can effectively improve the initial burst release of drugs [19], and the release behavior of drugs can be facilitated by increasing the hydrophilicity of fiber materials and the compatibility of drugs and fiber materials [20]. PCL is compatible with other organic polymers, easily soluble in a variety of organic solvents, and widely used in biomedical materials [21]. However, due to its poor hydrophilicity, pure PCL material is not conducive to cell adhesion and growth [22]. PEG has good hydrophilicity, biocompatibility, and no rejection reaction [23], and its appropriate addition can be used to enhance the hydrophilicity of materials. Bui et al. [24] prepared PCL/PEG nanofibers containing curcumin and found that the addition of PEG improved the spinning solution viscosity made the drug-loaded nanofibers have a porous surface and enhanced the biocompatibility of the nanofibers. Riaz et al. [25] introduced PEG into the preparation of PCL nanofibers, which not only reduced the fiber diameter but also improved the crystallinity and wettability of PCL nanofibers. Dehghan et al. [26] prepared PCL/PEG/aloe vera nanofiber scaffolds, and osteoblast-like cells showed good penetration, growth, and proliferation capabilities on them.

Simvastatin (SIM) is a liposoluble hydrophobic drug, which can promote the expression of vascular endothelial factor (VEGF) and bone morphogenetic protein (BMP-2) in vivo and in vitro, thus promoting the differentiation of osteoblasts and angiogenesis. Meanwhile, it can also inhibit the role of osteoclasts and be used to repair bone defects [27]. However, SIM is difficult to dissolve in water and its bioavailability is very low, which limits its role in promoting osteogenesis. Loading SIM into fibers by electrospinning can improve the biocompatibility of drug-loaded fiber materials by adjusting the drug release behavior [28] so that SIM can give full play to its role in promoting osteogenesis. Nevertheless, SIM drug particles have a large particle size, and are fat-soluble hydrophobic drugs, which have poor compatibility with fiber materials, cannot be dispersed evenly in the spinning solution, and cannot be effectively coated in the fibers, so some drugs will be exposed on the fiber surface and cause the initial burst release of drugs [29].

Stearic acid (SA) is a kind of fatty acid widely existing in nature, which has good biocompatibility and is often used to prepare liposome biomaterials [30]. In this study, based on our previous work [31], SIM was first dissolved in SA solution, and then SA/SIM solution was dropped into the PCL/PEG spinning solution for electrospinning, which could make SIM disperse more evenly in the spinning solution, improve the compatibility between SIM and materials, and enhance the morphology and performance of nanofiber membranes (NFMs). Therefore, the effects of the addition of SIM and SA on the morphology, structure, and properties of electrospun PCL/PEG NFMs were investigated, and the effects of SA addition on the mechanical properties, wettability, and drug release properties of SIM-loaded composite NFMs were further studied.

## 2. Materials and Methods

### 2.1. Materials

Polycaprolactone (PCL, *Mw* = 80000 Da) and penicillin-streptomycin (P&S) were purchased from Shanghai Yuanye Biochemical. Co., Ltd., Shanghai, China, Polyethylene glycol (PEG, *Mw* = 2000 Da) was obtained from Suzhou Sinopharm Co., Ltd., Suzhou, China, Stearic acid (SA, Analytical pure) and sodium dodecyl sulfonate (SDS) (purity ≥ 95%) were purchased from Aladdin Co., Ltd., Hongkong, China, Simvastatin (SIM, cell culture grade) was provided by Merrill Co., Ltd., New York, NY, USA, Tetrahydrofuran (THF) and anhydrous ethanol (AE) (analytically pure) were purchased from Jiangsu Qiangsheng Functional Chemistry Co., Ltd., Suzhou, China, N-butanol (Analytical Reagent) was purchased from Wuxi Zhanwang Chemical Reagent Co., Ltd., Wuxi, China, Phosphate buffer saline (PBS, PH 7.4) was purchased from Corning Co., Ltd., Glendale, CA, USA, Minimum essential medium-Alp (α-MEM) was purchased from Shanghai BasalMedia Technologies Co., Ltd., Shanghai, China, Fetal bovine serum (FBS) was purchased from Thermo Fisher Scientific, Waltham, MA, USA. Almar blue was purchased from Yeasen Biotechnology (Shanghai) Co., Ltd., Shanghai, China, Calcein acetoxy methyl ester (Calcein-AM) was purchased from Shanghai Beyotime Institute of Biotechnology, Shanghai, China. Mouse pre-osteoblasts (MC3T3-E1) were provided by Soochow University.

### 2.2. Preparation of Nanofiber Membrane

According to our previous work [31], a certain amount of PCL granulates and PEG flakes were dissolved in the mixed solvent of THF and AE with a ratio of 5:1, stirred with a magnetic stirrer (HJ-6A, Gongyi Yuhua Instrument Co., Ltd., Zhengzhou, China) at room temperature until uniform clarification to configure PCL/PEG solution with a total solute mass fraction of 25 wt%, in which the mass ratio of PCL:PEG was 8:2. Meanwhile, some SA granulates were dissolved in AE, and stirred in a water bath at 60 °C until SA solution was homogeneous and clear. After cooling to room temperature, a certain mass of SIM powders was added to the SA solution and stirred evenly to prepare SA/SIM solution with a 3 wt% mass fraction of SA and 5 wt% mass fraction of SIM. Finally, 0.16 g of SIM powders and 4 mL of SA/SIM solution were added separately to centrifuge tubes containing 20 mL PCL/PEG solution and uniformly dispersed by utilizing an ultrasonic machine (SL-5200DT, Nanjing Shunliu Instrument Co., Ltd., Nanjing, China) for 10 min to prepare SIM/PCL/PEG and SA/SIM/PCL/PEG solutions with equal drug content.

The configured three different spinning solutions were transferred to a 10 mL syringe separately for electrospinning, respectively. The syringe needle (18 G, 1.2 mm) was connected to the positive pole of the DC high-voltage power supply, while the stationary copper collector with silicon paper on the surface was connected to the negative pole of the DC high-voltage power supply. The electrospinning parameters were: the flow rate was 1 mL/h, the applied voltage was 15 kV, and the collecting distance was 15 cm. All electrospinning experiments were carried out at 25 °C and relative humidity of 55% for a certain period of time until the spinning solution was completely spun to prepare PCL/PEG NMFs, SIM/PCL/PEG NFMs and SA/SIM/PCL/PEG NMFs with a drug loading capacity of 0.16 g, respectively. The codes of NFMs obtained by electrospinning and their corresponding spinning solution components were shown in Table 1.

### 2.3. Characterization

The dynamic viscosity and electrical conductivity of spinning solutions were characterized by a viscometer (SNB-1, Shanghai Nirun Technology Co., Ltd., Shanghai, China) at a constant speed of 60 r/min and a conductivity meter (DDS-307A, Shanghai INESA Analytical Instrument Co., Ltd., Shanghai, China), respectively. The measurement was repeated five times at room temperature.

The morphology of NFMs was investigated by scanning electron microscopy (SEM, Hitachi S-4800, Tokyo, Japan). 10 SEM images and 50 nanofibers at random in each SEM image of every specimen were used for fiber diameter distribution analysis of NFMs by Image J software (National Institute of Mental Health, Bethesda, MD, USA).

The mixture of a crushed specimen and KBr powder was pressed into sheet-like specimens, which were characterized by a Fourier transform infrared (FTIR) spectroscopy (Perkin Elmer SpectrumGX, Shelton, CT, USA) with 64 scans and a wavenumber range of 500–4000 cm^−1^.

The specimens with a length of 4 cm and a width of 1 cm were prepared from different NFMs, and 10 specimens with the thickness measured and recorded were prepared for each NFM. And then the tensile test of each specimen was carried out, using a universal material testing instrument (INSTRON-3365, INSTRON, Norword, MA, USA) at the speed rate of 20 mm/min and with a clamping distance of 20 mm. The Young’s modulus of the specimen was expressed by the slope of its stress-strain curve, and the tensile strength of the specimen was calculated according to the following equation [32]:

$$specimen\;tensile\;strength\left(MPa\right)=\frac{specimen\;tensile\;force\left(N\right)}{specimen\;thickness\left(mm\right)*specimen\;width\left(mm\right)}$$

The water contact angle (CA) of different specimens was measured by a Krüss DSA100 apparatus (Krüss Company, Hamburg, Germany). The volume of droplet used for static CA was 6 μL. The average CAs were calculated by measuring 10 specimens for each NFM.

### 2.4. In Vitro Drug Release Test

The PBS solution containing SDS (0.5 wt%) was used as the in vitro release medium. The addition of SDS was to increase the solubility of hydrophobic SIM, which was conducive to its release in the medium [33]. Two kinds of the SIM-loaded NFMs (NO.1 and NO.2) were cut into circular specimens with a diameter of 15 mm. Five specimens were taken from each NFM and placed in a 50 mL centrifuge tube respectively. After adding 10 mL of release medium respectively, these centrifuge tubes were transferred to a shaking table (FLY-100/200, Shanghai Shenxian Thermostatic Equipment Factory, Shanghai, China) at 37 °C with a shaking speed of 60 rpm. At the required time interval, 2 mL of the release mediums were withdrawn for SIM release rate analysis, and the same amount of fresh release mediums was replenished for sustaining the incubation. The OD value of each release specimen at 239 nm was detected with an ultraviolet (UV) spectrophotometer (Cary 5000, Agilent, Santa Clara, CA, USA). And then the SIM release amount of each specimen at each time point was determined according to the calibration curve of SIM in the release medium, and the cumulative release rates of SIM-loaded NFMs were confirmed.

### 2.5. In Vitro Biocompatibility Test

Cell cultures were carried out using MC3T3-E1, which were cultured in complete medium (including 90 wt% α-MEM medium, 10 wt% FBS, and 1 wt% P&S). NFMs were cut into circular specimens with a diameter of 15 mm. The specimens were fumigated with methanol at 37 °C for 2 h, then transferred to an ultraviolet lamp for sterilization for 30 min, and then put in the sterile operation platform for air drying before being put into the bottom of a 24-well plate. Afterward, 1 mL of cell suspension with a certain concentration was vertically dropped into each well, and 5 specimens were set for each NFM. The 24-well plate with the cells planted was put into the incubator for culture, and the culture medium was updated every two days. Cells cultured on tissue culture polystyrene (TCPs) were used as controls.

Alamar blue, as a cell-proliferation assay, was used to detect the proliferation activity of MC3T3-E1 cells. The cells were seeded on the NFMs at a cell density of 1×10^4^ cells/well, placed in a cell incubator (37 °C, 5 % CO_2_), and incubated for 1, 3, 5, and 7 days. At the corresponding time, the 24-well plate was taken out, the medium in the well plate was aspirated, and 200 μL of Alamar blue solution (containing 90 wt% α-MEM medium and 10 wt% Alamar blue indicator) was added to each well, which was put into the cell incubator for 2 h. Afterward, 100 μL of the medium was shifted from each well to a black 96-well plate. The fluorescence value of the solution in the well plate was measured by a microplate reader (Synergy HT, Bio-Tek Instruments, Winooski, VT, USA), the wavelength was set to 590 nm, and the proliferation of living cells was investigated by a confocal laser scanning microscope (CLSM, Fv1000, Olympus, Tokyo, Japan).

## 3. Results and Discussion

### 3.1. Property Characterization of Spinning Solutions

**Figure 1:**
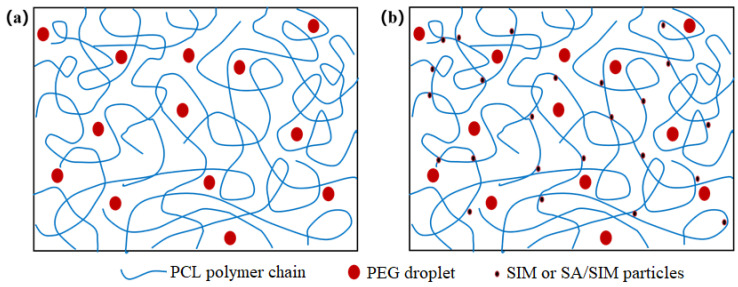
Molecular chain entanglement and molecular diagram in different spinning solutions (NO.0 (**a**), NO. 1 or NO.2 (**b**)).

When different polymers are mixed in a solvent, the polymer solution can behave as a mixed clear solution or an unmixed emulsion [34,35,36,37,38], while PCL/PEG solution is a clear solution, in which PEG is uniformly dispersed as small droplets in the continuous phase of PCL [39]. The viscosities and conductivities of the three spinning solutions were shown in Table 2. It could be found that the viscosities of NO.1 and NO.2 spinning solutions with SIM decreased, especially the viscosity of NO.2 spinning solution with SA/SIM solution decreased significantly and its conductivity increased obviously. This might be because, in the NO.0 spinning solution, the molecular chains of PCL were fully entangled, but PCL was incompatible with PEG-2000, which made PEG disperse in the continuous phase of PCL in the form of droplets. When SIM was added to the NO.0 spinning solution, the molecules of hydrophobic SIM were close to the dispersion phase of the same hydrophobic PCL under the van der Waals force and electrostatic force, resulting in their soft agglomeration and flowing together, thus playing the role of dredging and lubrication, as shown in Figure 1. This would dredge the entangled PCL molecular chains and reduce the entanglement degree of PCL molecular chain, thus reducing the viscosity of the NO.1 spinning solution. When SA/SIM solution was added to the NO.0 spinning solution, the entanglement degree of the PCL molecular chain was similarly reduced, and the overall concentration of the spinning solution was also reduced, resulting in a significant decrease in the viscosity of the NO.2 spinning solution. Moreover, the conductivity of the NO.2 spinning solution increased due to more free ions in the added SA/SIM solution.

### 3.2. Morphology Analysis of SIM and SA/SIM Particles

**Figure 2:**
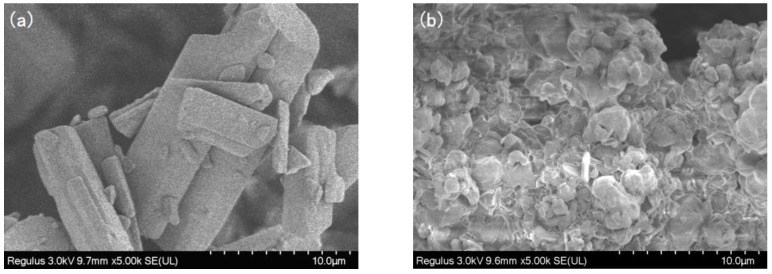
SEM images of SIM (**a**) and SA/SIM particles (**b**).

The SEM images of SIM and SA/SIM particles were shown in Figure 2. It could be found that after SIM particles were dissolved in the SA solution, the size of SA/SIM particles was significantly smaller, which was conducive to their dispersion in the spinning solution.

### 3.3. Morphology Characterization of NFMs

**Figure 3:**
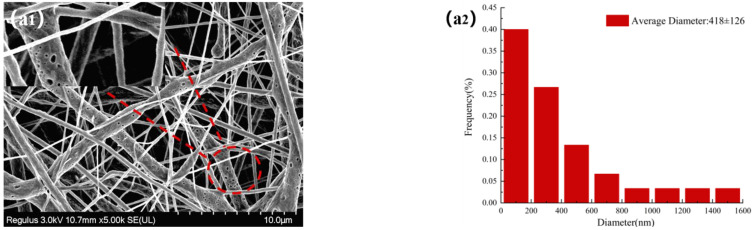

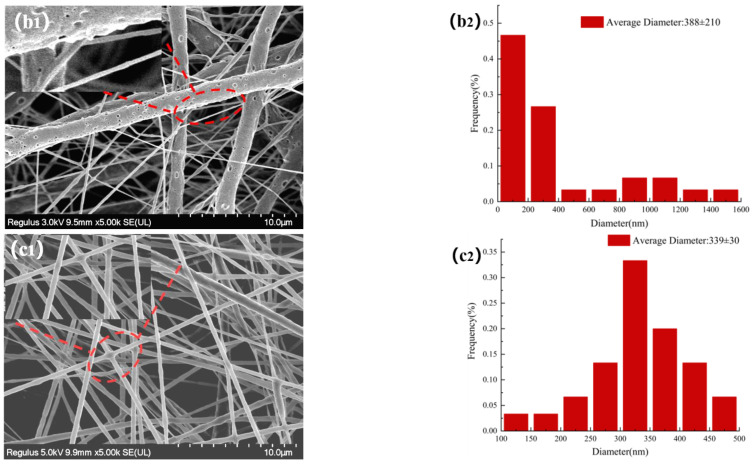
Morphology and corresponding diameter distribution of different NFMs (NO.0 (**a**), NO.1 (**b**), NO.2 (**c**)).

The morphologies of NFMs were investigated by SEM. Figure 3 displayed SEM images of NFMs and their corresponding nanofiber diameter distributions. As shown in Figure 3a, the nanofiber diameter distribution of NO.0 NFM was relatively uneven, and some fibers were curved and sticky. It was because of the slower evaporation of the solvent during the spinning process and the larger viscosity of NO.0 spinning solution, causing the polymer molecular chains excessively entangled and the poor fluidity of the spinning solution, which made some fibers unable to be effectively stretched under the action of electric field and bonded together. As shown in Figure 3b, the average fiber diameter of NO.1 NFM was smaller than that of NO.0 due to the relatively small viscosity of the NO.1 spinning solution. However, its fiber diameter distribution was more uneven, which was because the NO.1 spinning solution contained SIM particles with large size, which had poor solubility and uneven dispersion in the spinning solution, resulting in a large difference in fiber thickness. Furthermore, it could also be observed in Figure 3b that there were some tiny spikes on the fiber surface, which was caused by the fact that some SIM particles were too large to be completely coated. As shown in Figure 3c, the fiber morphology of NO.2 NFM was smoother and straighter, no tiny spikes be observed on the fiber surface, and its fiber diameter distribution was more uniform, which was mainly because of the smaller viscosity and higher conductivity of NO.2 spinning solution, and the smaller size of SA/SIM particles in the spinning solution. The size of SIM particles loaded on the nanofibers in Figure 3b was smaller than that in Figure 2a, which might be because, during the preparation of the spinning solution, SIM would be partially dissolved in the solution after full stirring, which made the size of the large SIM particles smaller.

### 3.4. Fourier Transform Infrared (FTIR) Analysis

**Figure 4:**
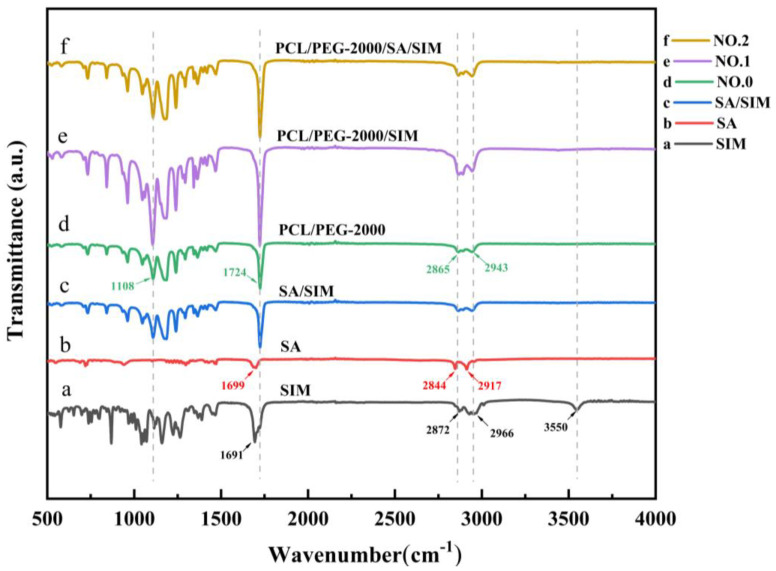
FTIR spectra of specimens.

The acquired FTIR spectra of SIM, SA, SA/SIM, and different NFMs were presented respectively in Figure 4. The characteristic peaks of SIM (Figure 4a) were the ester group (C=O) vibration at 1691 cm^−1^, symmetric C-H and asymmetric stretching vibrations at 2872 cm^−1^ and 2966 cm^−1^, and O-H stretching vibration at 3550 cm^−1^. The FTIR spectra of SA (Figure 4b) displayed the characteristic peck produced by C=O stretching vibration in fatty acids at 1699 cm^−1^, and O-H stretching vibration peaks of fatty acids at 2844 cm^−1^ and 2917 cm^−1^. The FTIR spectra of NO.0 NFM (Figure 4d) exhibited the C-O-H stretching vibration of PEG at 1108 cm^−1^, C=O stretching vibration of PCL at 1724 cm^−1^, and symmetric and asymmetric methylene stretching vibrations of PCL at 2865 cm^−1^ and 2943 cm^−1^. After adding SIM to SA and polymer (Figure 4c,e,f), the peak value of ester group (C=O) vibration of SIM moved to 1724 cm^−1^, and the peak value of O-H vibration almost disappeared at 3550 cm^−1^, indicating that there were SIM drugs in SA/SIM powders, NO.1 NFM and NO.2 NFM [40], and the SIM drugs were coated in the polymer. Meanwhile, there were no new characteristic peaks in SA/SIM, NO.1 and NO.2, illustrating that there was no chemical reaction between SA, PCL, PEG, and SIM in the process of mixing, dissolving, and spinning, which was a pure physical combination.

### 3.5. Mechanical Properties of NFMs

**Figure 5:**
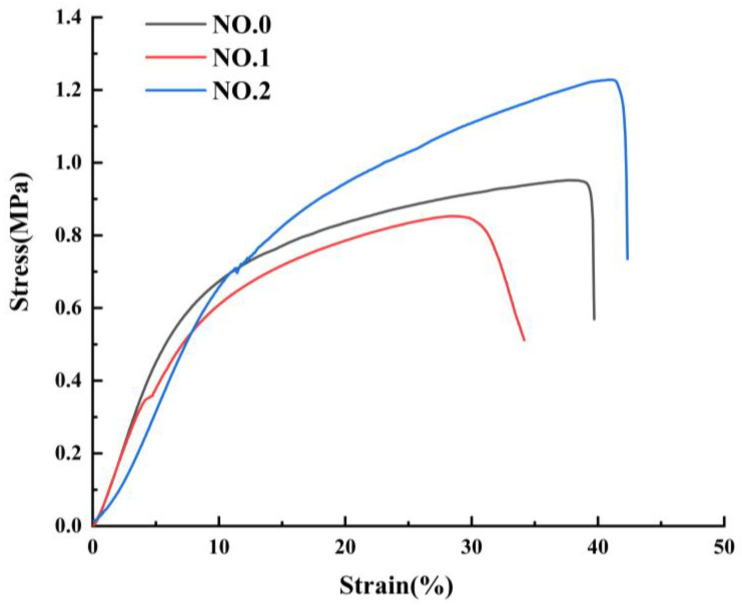
Mechanical properties of NFMs: stress-strain curve.

The mechanical properties of NFMs were characterized as shown in Figure 5 and Table 3. The tensile behavior exhibited by the three different NFMs is common, as reported in different literature [41,42,43]. It could be seen from Figure 5 that during the stretching process, the beginning of the curve was a straight line, where the elastic deformation was first triggered until the stress exceeded the elastic limit and entered the yielding phase. At this stage, the plastic deformation increased sharply, the curve growth became slow, and the stress and strain fluctuate slightly before the larger plastic stress caused the NFMs to fracture. The similar Young’s modulus of NO.0 and NO.1 NFMs could be observed in the initial segments of the curves, and Young’s modulus NO.2 NFM was smaller, which made it have a small stiffness. And the reduction in the stiffness of NO.2 NFM was likely due to the fact that the molecular chains of NO.2 NFM were less entangled and the consequent low cohesive forces made the material more deformable. In addition, NO.1 NFM had the smallest fracture strength and elongation at break, on account of the extremely uneven distribution of fiber diameters and the potentially larger SIM particles integrated into the fibers, causing the fracture at the weak section of the fibers. It was worth noting that the fracture strength of NO.2 NFM increased to 1.24 ± 0.03 MPa and its elongation at break increased to 43.89 ± 2.15% by appending SA/SIM particles, which benefited from the finer and uniformly distributed fiber diameter [44]. Besides, as a joint result of the homogeneous mixing of polymers in NO.2 NFM and the bonding points between the blended fibers [45], NO.2 NFM had the best tensile resilience and dimensional stability.

### 3.6. Wetting Properties of Nanofiber Membranes

**Figure 6:**
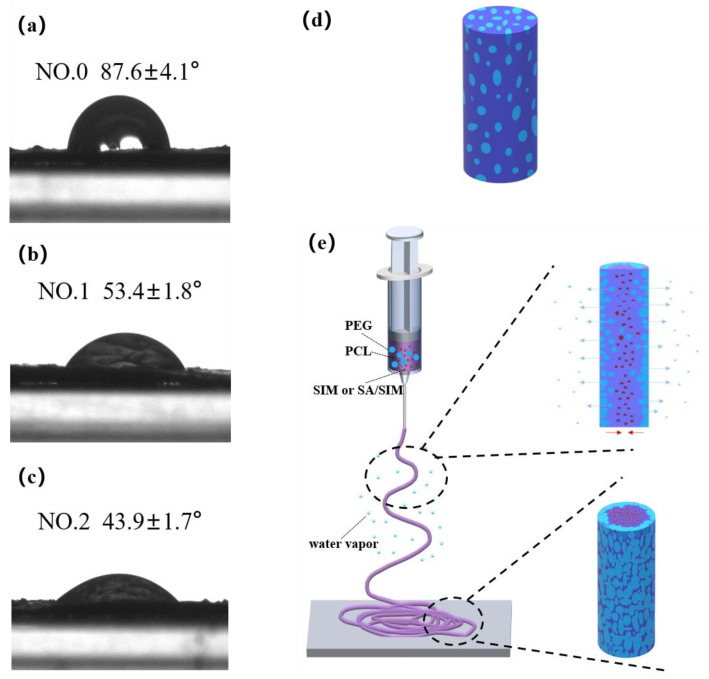
Contact angle test pictures of NFMs (NO.0 (**a**), NO.1 (**b**), NO.2 (**c**)), single fiber diagram of NO.0 (**d**) and schematic diagram of material migration during electrospinning (**e**).

The contact angle (CA) characterization of NFMs and schematic diagram of material migration during electrospinning were shown in Figure 6. It could be seen that the CA of NO.0 NFM was the largest, and the CA of NO.2 NFM was the smallest, indicating that the hydrophilicity of NO.2 NFM was the best. This was because NO.0 NFM was composed of hydrophobic PCL and hydrophilic PEG, which were evenly distributed in a single fiber, as shown in Figure 6d, but the mass fraction of PCL was larger than that of PEG, resulting in the relatively poor hydrophilicity of NO.0 NFM. Compared with NO.0 NFM, NO.1 and NO.2 NFMs contained hydrophobic SIM and SA/SIM respectively, but their hydrophilicity was significantly improved. This might be because during the spinning process, under the action of electrostatic force and van der Waals force, hydrophobic SIM or SA/SIM were soft agglomerated with hydrophobic PCL, making hydrophobic substances agglomerate inside the fiber, while PEG with good hydrophilicity tended to migrate to the environment with high humidity outside the fiber, and finally dispersed outside the fiber. The process was shown in Figure 6e. In addition, since the CA increased with the increase of the roughness of the material surface [46], the hydrophilicity of NO.2 NFM with a smooth surface was better than that of NO.1 NFM.

### 3.7. Analysis of Drug Release Performance

**Figure 7:**
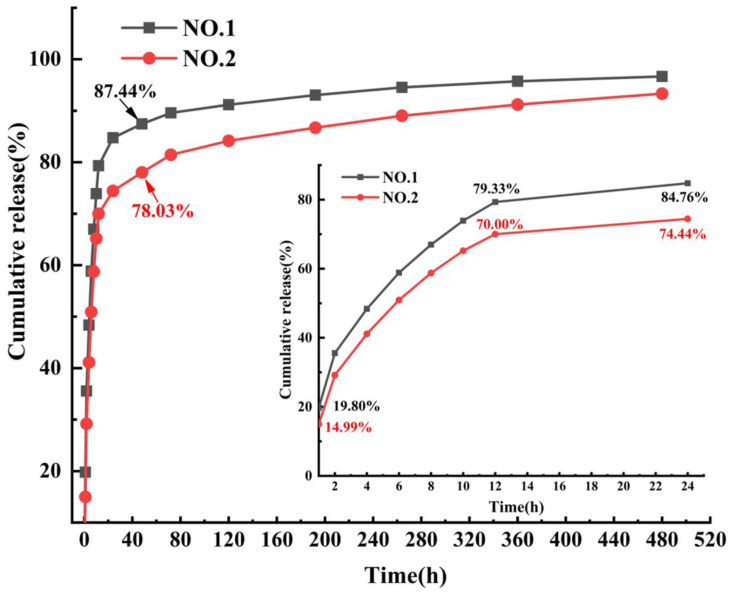
SIM release curves of NO.1 and NO.2 NFMs.

The SIM release curves of NO.1 and NO.2 NFMs were shown in Figure 7. It was found that the drug release rate of NO.1 NFM was 19.80% within 1 h, and its cumulative release rate reached 84.76% at the 24th hour. In contrast, the cumulative drug release rate of SIM/polyethylene oxide NFM reached 98.86% within 12 h [30], which showed that as the carrier of SIM, the PCL/PEG hybrid phase was conducive to alleviating the phenomenon of initial burst release of drugs and achieving a longer-term sustained drug release. After adding SA/SIM, compared with those of NO.1 NFM, the cumulative drug release rates of NO.2 NFM at 1 and 24 h were reduced to 14.99% and 74.44%, respectively, and the initial burst release of drugs was significantly further alleviated. Since the drug release behavior mainly follows the classical Fick’s law, the drug released from the initial stage of the two drug-loaded NFMs is mainly from the drug particles distributed or exposed on the fiber surface. However, NO.1 NFM was fabricated by directly adding SIM into the PCL/PEG spinning solution, and the poor solubility of SIM particles in the PCL/PEG solution made them unevenly dispersed in the spinning solution. Accordingly, some large particles of SIM were not effectively coated, which accelerated the drug release rate and produced the initial burst release of drugs. While NO.2 NFM used SA solution to increase the solubility of SIM, and reduced the particle size of SIM, making it evenly distributed in the spinning solution and finally effectively coated in the fiber. Therefore, NO.2 NFM could alleviate the initial burst release of drugs.

### 3.8. Biocompatibility of NMFs

**Figure 8:**
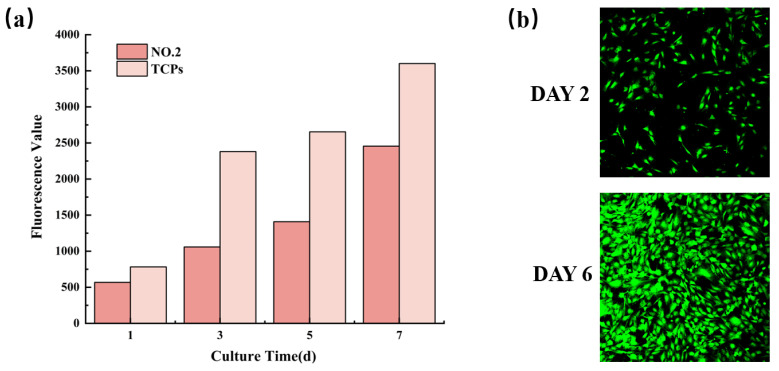
Proliferation activity (**a**) and CLSM micrographs (**b**) of MC3T3-E1 cells on NO.2 NFM.

The biocompatibility characterization of NO.2 NFM was shown in Figure 8. It could be observed from Figure 8a that the fluorescence values of cells on NO.2 NFM increased with time, which revealed that MC3T3-E1 cells continued to proliferate in the NFM, indicating the good biocompatibility of NO.2 NFM. Moreover, Figure 8b displayed the CLSM micrograph of MC3T3-E1 cells cultured on No.2 NFM for 2 and 6 days, which further proved that No.2 NFM had good biocompatibility.

## 4. Conclusions

In this study, the combination of PCL and PEG became a hybrid biomaterial composite with acceptable mechanical properties and good biocompatibility. SA with good biocompatibility was innovatively added to increase the dispersion uniformity of SIM in the spinning solution, thus improving the compatibility between SIM and polymers, and enhancing the morphology and performance of SIM-loaded PCL/PEG NFMs. Accordingly, the effects of the addition of SIM and SA on the spinning solution properties were investigated, and the effects of SA addition on the morphologies, mechanical properties, wettability, and drug release properties of SIM-loaded composite NFMs were further studied. The results showed that after SIM was dissolved in SA solution, the particle size of SIM was significantly reduced and could be evenly dispersed in the polymer spinning solution, which made NO.2 spinning solution have the smallest viscosity and the highest conductivity. Therefore, NO.2 NFM had the best fiber morphology, the most uniform fiber diameter distribution, the optimum mechanical properties and the relative best hydrophilicity. The initial drug burst release from NO.1 NFM was alleviated during the drug release from NO.2 NFM, and both of the two drug-loaded NFMs had reached sustained release for more than 20 days, suggesting that NO.2 NFM might be a potential candidate in the application of drug delivery system. In addition, the proliferation of MC3T3-E1 cells on NO.2 NFM exhibited good biocompatibility. However, compared with TCPs, NO.2 NFM did not significantly promote cell growth, which would limit its application in tissue engineering. In our future work, the methods and processes of preparing SA/SIM loaded PCL/PEG NFMs with other fiber structures (such as core-shell structures) will be explored to make them more suitable for tissue engineering.

## Figures and Tables

**Table 1:** Codes of electrospun NFMs and their corresponding spinning solution components.

Electrospun NFM Code	Spinning Solution Composition
NO.0	25 wt% PCL/PEG (PCL:PEG = 8:2)
NO.1	25 wt% PCL/PEG (PCL:PEG = 8:2) 0.16 g SIM
NO.2	25 wt% PCL/PEG (PCL:PEG = 8:2) 5 wt% SIM/3 wt% SA containing 0.16 g SIM

**Table 2:** Viscosities and conductivities of different spinning solutions.

Spinning Solution Code	Viscositiy (mPa.S)	Conductitivity (μS/cm)
NO.0	307	7.79
NO.1	275	8.15
NO.2	126	14.19

**Table 3:** Tensile properties of NFMs.

NFM	Thickness (mm)	Young’s Modulus (MPa)	Fracture Strength (MPa)	Elongation at Break (%)
NO.0	0.11 ± 0.03	0.10 ± 0.09	0.93 ± 0.05	35.27 ± 3.98
NO.1	0.16 ± 0.04	0.09 ± 0.07	0.85 ± 0.09	31.04 ± 1.76
NO.2	0.11 ± 0.03	0.06 ± 0.07	1.24 ± 0.03	43.89 ± 2.15

## Data Availability

Not applicable.
